# Development and Validation a Nomogram Incorporating CT Radiomics Signatures and Radiological Features for Differentiating Invasive Adenocarcinoma From Adenocarcinoma *In Situ* and Minimally Invasive Adenocarcinoma Presenting as Ground-Glass Nodules Measuring 5-10mm in Diameter

**DOI:** 10.3389/fonc.2021.618677

**Published:** 2021-04-21

**Authors:** Lili Shi, Weiya Shi, Xueqing Peng, Yi Zhan, Linxiao Zhou, Yunpeng Wang, Mingxiang Feng, Jinli Zhao, Fei Shan, Lei Liu

**Affiliations:** ^1^Shanghai Public Health Clinical Center and Institutes of Biomedical Sciences, Fudan University, Shanghai, China; ^2^Medical School, Nantong University, Nantong, China; ^3^Chest Surgery Department, Zhongshan Hospital, Fudan University, Shanghai, China; ^4^Radiology Department, Affiliated Hospital of Nantong University, Nantong, China; ^5^School of Basic Medical Sciences, and Academy of Engineering and Technology, Fudan University, Shanghai, China

**Keywords:** ground-glass nodules, computed tomography, radiomics, lung cancer, invasive adenocarcinoma

## Abstract

**Purpose:**

To develop and validate a nomogram for differentiating invasive adenocarcinoma (IAC) from adenocarcinoma *in situ* (AIS) and minimally invasive adenocarcinoma (MIA) presenting as ground-glass nodules (GGNs) measuring 5-10mm in diameter.

**Materials and Methods:**

This retrospective study included 446 patients with 478 GGNs histopathologically confirmed AIS, MIA or IAC. These patients were assigned to a primary cohort, an internal validation cohort and an external validation cohort. The segmentation of these GGNs on thin-slice computed tomography (CT) were performed semi-automatically with in-house software. Radiomics features were then extracted from unenhanced CT images with PyRadiomics. Radiological features of these GGNs were also collected. Radiomics features were investigated for usefulness in building radiomics signatures by spearman correlation analysis, minimum redundancy maximum relevance (mRMR) feature ranking method and least absolute shrinkage and selection operator (LASSO) classifier. Multivariable logistic regression analysis was used to develop a nomogram incorporating the radiomics signature and radiological features. The performance of the nomogram was assessed with discrimination, calibration, clinical usefulness and evaluated on the validation cohorts.

**Results:**

Five radiomics features remained after features selection. The model incorporating radiomics signatures and four radiological features (bubble-like appearance, tumor-lung interface, mean CT value, average diameter) showed good calibration and good discrimination with AUC of 0.831(95%CI, 0.772~0.890). Application of the nomogram in the internal validation cohort with AUC of 0.792 (95%CI, 0.712~0.871) and in the external validation cohort with AUC of 0.833 (95%CI, 0.729-0.938) also indicated good calibration and good discrimination. The decision curve analysis demonstrated that the nomogram was clinically useful.

**Conclusion:**

This study presents a nomogram incorporating the radiomics signatures and radiological features, which can be used to predict the risk of IAC in patients with GGNs measuring 5-10mm in diameter individually.

## Introduction

Lung cancer is one of the most commonly diagnosed human malignancy and the leading cause of cancer-related death worldwide (1). Adenocarcinoma is the most common histologic type of lung cancer and its incidence has increased over the past few decades, accounting for more than 40% of the total nowadays (2). It was classified into atypical adenomatous hyperplasia (AAH), adenocarcinoma *in situ* (AIS), minimally invasive adenocarcinoma (MIA) and invasive adenocarcinoma (IAC) in the 2015 World Health Organization (WHO) classification of lung tumors (3). Patients with IAC have a higher risk of recurrence and are usually treated with lobectomy (4). Nowadays, segmentectomy is suggested for selected patients with clinical N0 IAC of no more than 2cm in diameter (4). Patients with AIS or MIA(AIS/MIA) are managed with active surveillance or sublobar resection because of excellent prognosis (5). The subtypes of adenocarcinoma are currently determined mainly by biopsy or postoperative pathological sections in clinical practice, which are invasive and risky. Discriminating IAC from AIS/MIA before surgery could help clinicians to assess prognosis in order to improve clinical decision making and avoid over- or undertreatment, without the need for invasive procedures.

Adenocarcinoma frequently presents as pulmonary nodules including ground-glass nodules (GGNs) and solid nodules on computed tomography (CT). Radiological features such as air bronchogram, margin, pleural indentation have been found to be related with the malignancy or tumor histology of GGNs (6–8). These features are subjective, qualitative, and sometimes are not easily to be determined in small nodules with a diameter less than 10mm. Nodules with diameter less than 5mm are usually benign (9), however, some nodules less than 10mm have been pathologically confirmed as IAC (10).

Radiomics refers to high-throughput extraction of large amounts of image features from radiographic images (11, 12). Radiomics features can be calculated by computational methodologies to quantify the characteristics of tumor tissues and provide a detailed and comprehensive characterization of the tumor phenotype (13). Compared with conventional biomarkers, radiomics-based features are three-dimensional and the process of image acquisition is easy to perform, non-invasive and cost-effective. Several studies have shown the value of radiomics-based features in differentiating tumor subtypes by using different medical imaging modalities such as CT (14), magnetic resonance imaging (MRI) (15, 16) and positron emission tomography (PET) (17). Radiomics biomarkers have also been shown to be associated with several clinical events or endpoints, including tumor diagnosis (benign/malignant) (18), tumor subtyping (19), treatment response (20), patient survival (21), tumor recurrence and distant metastasis (22), tumor gene expression (23).

The purpose of this study was to investigate the ability of CT radiomics features combined with CT radiological features to differentiate IAC from AIS/MIA, and develop a nomogram incorporating CT radiomics signatures and radiological features to provide an individual, preoperative assessment of the risk of IAC in patients with GGNs measuring 5-10 mm.

## Materials and Methods

### Patient Cohort

Surgical datasets of three hospitals were reviewed. Patients were selected if they presented as lung nodules on chest CT scans and were diagnosed as pulmonary adenocarcinomas on the basis of pathologic analysis of surgical specimens. The nodules with the histopathological results AIS, MIA or IAC and the average diameter of nodule between 5mm and 10mm in CT scans were included. The exclusion criteria were as follows: 1) no routine CT examination had been performed in the month before surgery; 2) a series of consecutive CT images with a thickness of more than 1 mm; 3) CT images with severe respiratory motion artifacts; 4) the average diameter of nodule was smaller than 5mm or larger than 10mm; 5) the nodule presenting as a solid nodule. Some patients may have more than one nodule. These nodules were analyzed independently because they may be of different types.

**Figure 1:**
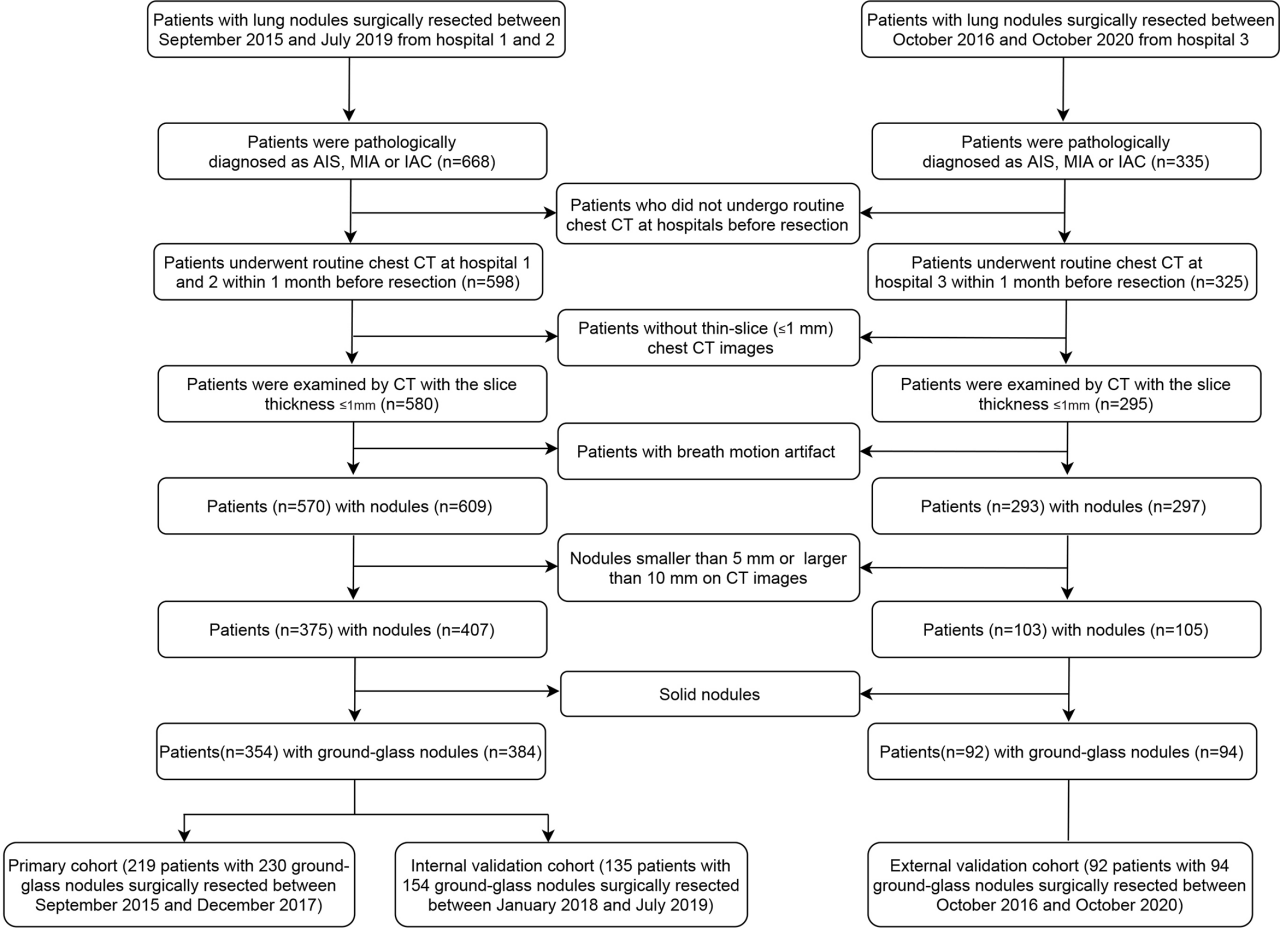
Flowchart of patient selection procedure.

A total of 354 eligible patients from hospital 1 and 2 were included, 219 patients with 230 GGNs between September 2015 and December 2017 in the primary cohort and 135 patients with 154 GGNs between January 2018 and July 2019 in the internal validation cohort. A total of 92 patients with 94 GGNs in hospital 3 between October 2016 and October 2020 were included in the external validation cohort. The flowchart of patient selection is listed in **Figure 1**. The study was approved by the institutional review boards of participating hospitals.

### Image Acquisition

An unenhanced chest CT examination was performed to obtain a whole lung scan in each patient. All patients were scanned while breath holding after deep inspiration. The CT images were obtained by any of four CT scanners, i.e., Brilliance 64 (Philips Medical Systems Inc., Netherlands), SOMATOM Definition AS (Siemens AG, Munich, Germany), SCENARIA (Hitachi Ltd., Tokyo, Japan), or Aquilion One (Canon Medical Supply Co., Ltd, Tokyo, Japan). The CT scan parameters of the above devices were as follows: tube voltage 120-130 kV; tube current 100-150 mAs; rotation time 0.5-0.75s; pitch 0.828-1.2; matrix 512*512 and standard resolution algorithms; reconstruction kernel of lung window, standard(B) (Brilliance 64), B60f sharp (SOMATOM Definition AS), Lung Sharp (SCENARIA), Lung-Std Axial (Aquilion One); lung window settings (width/level) 1200/-600 HU; mediastinal window settings (width/level) 400/40 HU; voxel dimensions 1×1×1-mm. The lung algorithm was used to reconstruct 1mm-thick sections of CT images.

### Radiological Features Extraction and Radiomics Features Extraction

Several radiological features were recorded. Two quantitative features were average diameter (defined as the mean of the longest diameter of the nodule and its perpendicular diameter at the same maximum axial slice) and mean CT value (measured for a sufficiently large round or oval regions of interest within the nodule on the maximum axial section). Qualitative features included nodule location (right upper lobe, right middle lobe, right lower lobe, left upper lobe, left lower lobe), type of nodule (pure GGN or mixed GGN), bubble-like appearance, pleural indentation, air bronchogram, pulmonary blood vessel change, margin defined as lobulation, spiculation or tumor-lung interface. Bubble-like appearance was defined as air-attenuated, vesicle-like lucency within the nodule. Air bronchogram sign was defined as the presence of ladiolucent bronchi within lesions. Vessels convergence or vessels dilatation within GGNs indicated pulmonary blood vessel change. Lobulation was defined when a portion of the nodule’s surface showed a wavy or scalloped configuration. Spiculation was defined as the presence of strands extending from the margin of the nodule into the lung parenchyma without reaching the pleural surface. Tumor-lung interface was recorded as clear if the nodules were well-defined.

The measurements were performed by two radiologists with more than 5 years of experience in chest radiology. The two radiologists measured each imaging feature independently, and the difference was reevaluated by the third radiologist with more than 20 years of experience in chest radiology. Any disagreements were resolved by consensus.

Nodule segmentation was performed semi-automatically with in-house software (24) and manually reviewed slice by slice by a radiologist with 6 years of experience in chest CT imaging and confirmed by another radiologist with 20 years of experience. A slice example of the nodule segmentation was provided in supplementary figure 1. After nodule segmentation, radiomics features were extracted from each nodule with open source PyRadiomics software (https://pyradiomics.readthedocs.io/en/latest/index.html) using the default settings. The software automatically calculated radiomics features for each included nodule.

### Radiomics Feature Selection

A total of 1525 features were extracted from CT images (the detailed radiomics features list was described in supplementary material). First, the variance of features close to 0 were removed. Pair-wise Spearman correlation analysis was performed to identify the redundant features. Features with the mean absolute correlation higher than 0.9 was considered redundant and eliminated. Then, a multivariable ranking method (minimum redundancy maximum relevance [mRMR]) was used to identify the most important features based on a heuristic scoring criterion, and only the top-ranked features were kept. Next, the top-ranking radiomics features were input into the least absolute shrinkage and selection operator (LASSO), which is suitable for regression of high-dimensional data, to obtain the optimal subset of radiomics features to build the radiomics signature for the evaluation of IAC and AIS/MIA. The receiver operating characteristic curve (AUC) was plotted versus log(λ) in order to identify the optimal value of log(λ). The optimal value was identified by the minimum criterion. The radiomics score (rad-score) of each GGN was calculated *via* a linear combination of selected features that were weighted by their respective coefficients.

### Model Building and Performance Assessment

The significance of associations with IAC and AIS/MIA was evaluated using the Fisher exact test for qualitative features and Mann-Whitney U test for mean CT value and average diameter. Two-sided p<0.1 was considered to indicate significant difference for qualitative features and p<0.05 for quantitative features.

The significantly different radiological features between the IAC group and the AIS/MIA group in the primary cohort combined with rad-score were included in the subsequent multivariable logistic regression analysis. Forward and backward step-wise selection was applied using the likelihood ratio test. We determined the optimal combinations of the features using the AKaike information criterion (AIC) (25). A nomogram was then constructed based on the multivariable logistic model. The discrimination of the nomogram was assessed with the AUC and validated in two validation cohorts. The calibration curves were used to assess the calibration of the nomogram. The goodness-of-fit of the nomogram was assessed with the Hosmer-Lemeshow test.

### Clinical Usefulness of Nomogram

To evaluate the potential clinical diagnostic effects of the nomogram model, a decision curve analysis was performed, which quantified the net benefits of using such a model at different threshold probabilities.

### Statistical Analysis

Statistical analyses were conducted with R software (version 3.6.3). The spearman correlation analysis was performed using the “caret” package. LASSO logistic regression was performed using the “glmnet” package. Logistic regression, nomogram construction and calibration plots were performed using the “rms” package. The decision curve was plotted using the “rmda” package. The Hosmer-Lemeshow test was done with the “vcdExtra” package. The ROCs were plotted and the DeLong test was used for pairwise comparisons between models using the “pROC” package. A two-sided p value <0.05 was considered significant.

## Results

### Patients’ Characteristics

Patients’ basic characteristics and nodule information in the primary and the validation cohorts are listed in **Table 1**. There were no statistically significant differences in gender distribution and age group between the primary cohort and the internal validation cohort, or between the primary cohort and the external validation cohort. Spiculation, lobulation, air bronchogram, and pulmonary blood vessel change didn’t show statistically significant difference between the IAC group and the AIS/MIA group either in the primary cohort or two validation cohorts. Mixed GGN and bubblelike appearance were significantly more common in the IAC group both in the primary cohort and two validation cohorts. Average diameter and mean CT value were significantly higher in the IAC group both in the primary cohort and two validation cohorts.

**Table 1:** Characteristics of the patients in the primary cohort and validation cohorts.

Variable	Primary cohort (n=230)			Internal validation cohort (n=154)			External validation cohort (n=94)			
	AIS/MIA (n=166)	IAC (n=64)	p-value	AIS/MIA (n=116)	IAC (n=38)	p-value		AIS/MIA (n=69)	IAC (n=25)	p-value
**Gender**			0.175			0.823				0.600
Male	37	20		25	9			18	5	
Female	129	44		91	29			51	20	
**Age (years)**			0.115			0.025				0.313
≦40	27	11		28	6			15	2	
40~65	124	41		74	20			46	20	
≥65	15	12		14	12			8	3	
**Type of nodule**			<0.001			<0.001				0.002
PGGN	130	28		86	15			54	11	
MGGN	36	36		30	23			15	14	
**Location**			0.479			0.002				0.917
Left upper lobe	47	18		32	13			11	4	
Left lower lobe	27	11		16	5			11	4	
Right upper lobe	46	22		32	18			16	6	
Right middle lobe	12	6		9	2			6	3	
Right lower lobe	34	7		27	0			9	3	
**Lobulation**			0.233			0.692				0.130
No	69	21		37	14			51	14	
Yes	97	43		79	24			18	11	
**Spiculation**			0.883			0.575				0.156
No	88	35		51	19			57	17	
Yes	78	29		65	19			12	8	
**Tumor-lung interface (clear)**			0.086			0.039				0.598
No	35	21		20	13			17	8	
Yes	131	43		96	25			52	17	
**Bubblelike appearance**			0.062			0.056				0.047
No	139	46		99	27			58	16	
Yes	27	18		17	11			11	9	
**Air bronchogram**			0.126			0.565				0.475
No	112	36		74	22			62	21	
Yes	54	28		42	16			7	4	
**Plumonary blood vessel change**			0.314			1.000				0.467
No	45	13		22	7			27	7	
Yes	121	51		94	31			42	18	
**Pleural indentation**			0.044			0.080				0.261
No	138	45		93	25			57	18	
Yes	28	19		23	13			12	7	
**Average Diameters (mm)**	7.34 ± 1.38	8.08 ± 1.03	<0.001	7.39 ± 1.30	8.34 ± 1.17	<0.001		7.86 ± 1.19	8.52 ± 1.03	0.012
**Mean CT value (HU)**	-534.73 ± 127.15	-393.86 ± 168.02	<0.001	-522.95 ± 152.19	-424.04 ± 147.42	<0.001		-537.74 ± 112.66	-409.25 ± 136.30	<0.001
**Rad-score**	-1.32 ± 0.69	-0.46 ± 0.75	<0.001	-1.17 ± 0.72	-0.49 ± 0.68	<0.001		-0.94 ± 0.63	-0.24 ± 0.67	<0.001

AIS, adenocarcinoma in situ; MIA, minimally invasive adenocarcinoma; IAC, invasive adenocarcinoma; PGGN, pure ground-glass nodule; MGGN, mixed ground-glass nodule; HU, Hounsfield units; Rad-score, radiomics score.

### Radiomics Features Selection and Radiomics Model Building

**Figure 2:**
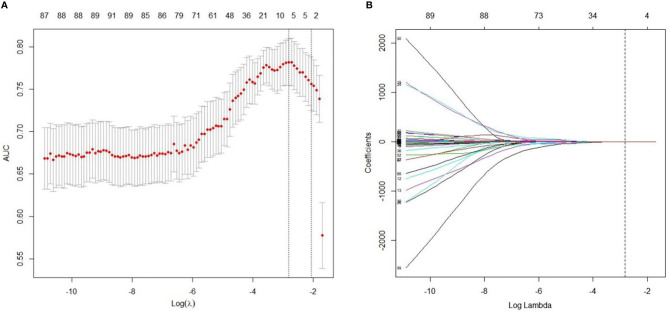
Radiomics feature selection using LASSO regression model. **(A)** Optimal feature selection according to AUC (area under curve) value. The dotted vertical lines were plotted at the optimal λ values based on the minimum criteria and 1 standard error of the minimum criteria. The optimal λ was selected. **(B)** LASSO coefficient profiles of the 100 radiomics features. Vertical line was drawn at the selected value using 10-fold cross-validation, where optimal λ resulted in five non-zero coefficients.

A total of 97 features with variance close to 0 were removed. Subsequently, after pair-wise spearman analysis, 246 features with the mean absolute correlation less than 0.9 remained. These features were ranked by mRMR, and then the top 100 features were selected. The LASSO classifier was trained on the primary cohort using the top 100 features. Five features with nonzero coefficients in the LASSO logistic model were selected (**Figure 2**). The rad-score was calculated for each patient based on the formula presented in the supplementary material.

The patients in the IAC group generally had higher rad-score than that in the AIS/MIA group in primary cohort (-0.46 ± 0.75 vs -1.32 ± 0.69, p<0.001), internal validation cohort (-0.49 ± 0.68 vs -1.17 ± 0.72, p<0.001) and external validation cohort (-0.24 ± 0.67 vs -0.94 ± 0.63, p<0.001). The AUC of radiomics model was 0.805 (95%CI, 0.741-0.869) with 75.0% sensitivity and 78.9% specificity in the primary cohort, 0.753 (95%CI, 0.666-0.841) with 63.2% sensitivity and 75.9% specificity in the internal validation cohort and 0.792 (95%CI, 0.680-0.904) with 72.0% sensitivity and 85.5% specificity in the external validation cohort.

### Nomogram Model Building, Assessment, and Validation

The radiological features that showed significant difference in univariate analyses in the primary cohort were included in multivariable logistic regression analysis. The predictors associated with IAC were bubble-like appearance, tumor-lung interface, mean CT value and average diameter.

**Figure 3:**
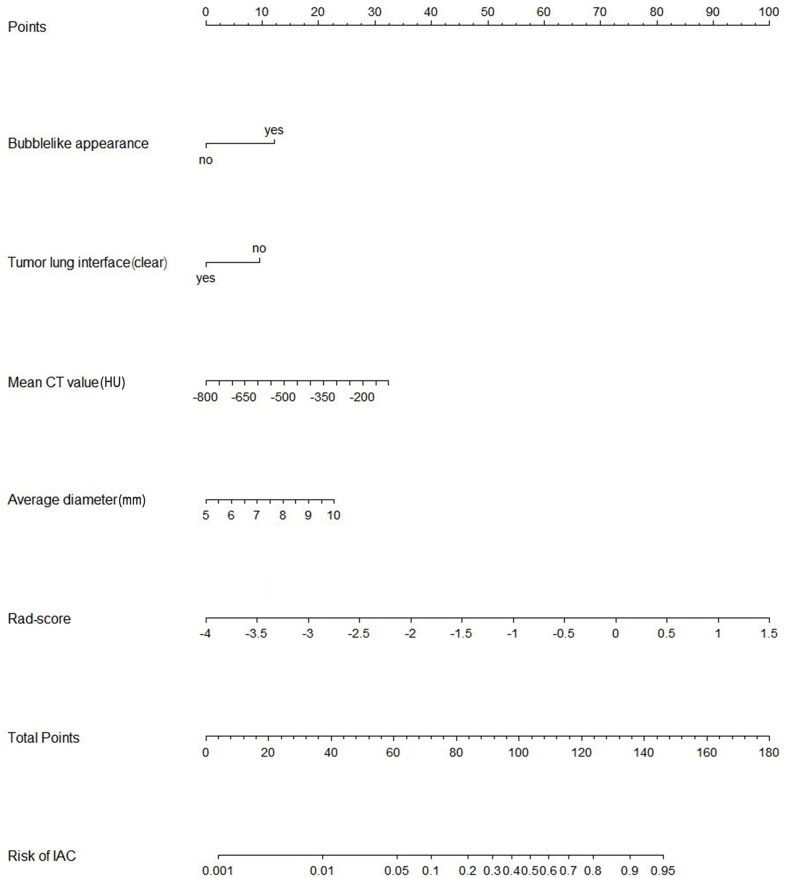
Nomogram of the model combining radiomics signatures and radiological features for predicting the risk of invasive adenocarcinoma. IAC, invasive adenocarcinoma; Rad-score, radiomics score.

**Figure 4:**
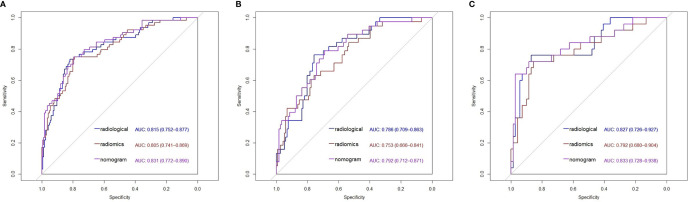
Receiver operating characteristic (ROC) curves of the radiological features model, radiomics features model and nomogram model in the primary cohort **(A)**, the internal validation cohort **(B)** and the external validation cohort **(C)**. AUC, Area under curve.

A nomogram model that incorporated these predictors and rad-score was developed (**Table 2**) and presented as the nomogram (**Figure 3**). The nomogram model yielded an AUC of 0.831 (95%CI, 0.772-0.890) in the primary cohort (**Figure 4A**), 0.792 (95%CI, 0.712-0.871) in the internal validation cohort (**Figure 4B**) and 0.833 (95%CI, 0.728-0.938) in the external validation cohort (**Figure 4C**). This model outperformed the radiomics signatures model and radiological features model both in primary cohort and two validation cohorts, though there was no statistically significant difference neither between nomogram model and radiological features model (p=0.225 in the primary cohort, p=0.778 in the internal validation cohort and p=0.785 in the external validation cohort) nor between nomogram model and radiomics signatures model (p=0.568 in the primary cohort, p=0.218 in the internal validation cohort and p=0.600 in the external validation cohort).

**Table 2:** Independent predictors identified in multivariable logistic regression.

Feature	OR	95%CI	p-value
(Intercept)	0.610	0.044~8.234	0.710
Bubble-like appearance	2.333	1.036~5.271	0.040
Tumor-lung interface (clear vs not clear)	0.518	0.237~1.130	0.097
Mean CT value	1.003	1.000~1.007	0.071
Average diameter	1.371	1.015~1.871	0.042
Rad-score	3.525	1.630~8.255	0.002

OR, odds ratio; CI, confidence interval; Rad-score, radiomics score.

**Figure 5:**
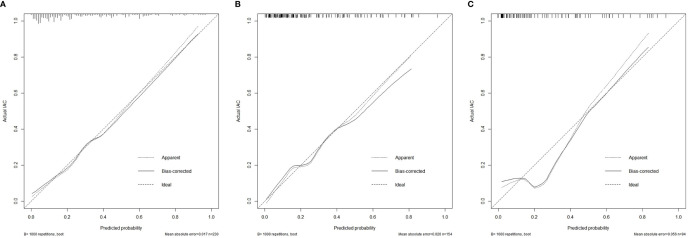
Calibration curves of the nomogram model showing the predicted versus actual probability for invasive adenocarcinoma in the primary cohort **(A)**, the internal validation cohort **(B)** and the external validation cohort **(C)**. IAC, invasive adenocarcinoma.

The calibration curves of the nomogram are shown in **Figure 5**. The Hosmer-Lemeshow test yielded a nonsignificant p value in the primary cohort, 0.225 in the internal validation cohort and 0.115 in the external validation cohort, which indicated good calibration power.

### Clinical Usefulness of the Nomogram

**Figure 6:**
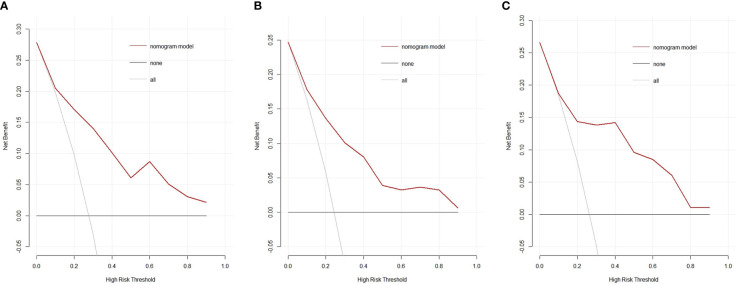
Decision curves of the nomogram model for predicting the risk of invasive adenocarcinoma in the primary cohort **(A)**, the internal validation cohort **(B)** and the external validation cohort **(C)**. The black line represents the assumption that no patients have IAC. The gray line represents the assumption that all patients have IAC. The red line represents the net benefit of using the nomogram model to predict IAC. The decision curve demonstrates that if the threshold probability is >10%, using the nomogram for IAC prediction adds more benefit than predicting either all or no patients. IAC: invasive adenocarcinoma.

The decision curve analysis showed that the nomogram had a higher overall net benefit, which indicated that the nomogram was clinically useful (**Figure 6A**). With a threshold probability of 10%, use of the nomogram could provide an added net benefit compared to the “treat-all” or “treat-none” strategy. Moreover, the similar findings were also observed in the internal validation cohort (**Figure 6B**) and the external validation cohort (**Figure 6C**).

## Discussion

We developed and validated a nomogram incorporating radiomics signature and radiological features for individualized preoperative predicting the risk of IAC in patients with GGNs measuring 5-10mm. The results showed that the discrimination and calibration of the nomogram model was favorable. This study provided a non-invasive preoperative prediction tool to identify patients with GGNs in a high risk of IAC.

The nomogram model finally incorporated the rad-score, based on five radiomics features, and four radiological features, according to AIC. Bubble-like appearance was more common in the IAC group than that in the AIS/MIA group, which was also found in the former study (26). Nodule diameter has always been considered as an important indicator in nodule management. Our model also identified the average diameter as an independent predictor for IAC prediction. In our study, clear tumor-lung interface was more common in the AIS/MIA group than that in the IAC group both in the primary cohort and the internal validation cohort, which was contrary to two previous studies (27, 28). The study by Wu et al. (27) included atypical adenomatous hyperplasia as preinvasive lesion. If atypical adenomatous hyperplasia was excluded, there was no significant difference in terms of tumor-lung interface between the IAC group and the AIS/MIA group, which was consistent with that in our external validation cohort. The study by Jin et al. (28) included nodules with diameter less than 30mm, while the diameter of nodules in our study was between 5-10mm. In addition, both studies (27, 28) included pure GGN only. Further studies are needed to confirm the relationship between tumor-lung interface and the invasiveness of lung adenocarcinoma. Mean CT value was higher in the IAC group in our study, which was consistent with the study by She et al. (29). Increased mean CT value reflected the increased heterogeneity of GGN (30). Zhao et al. (31) constructed a model included radiomics signature and mean CT value to predict the invasiveness of nodules. Another study (32) demonstrated that the AUC of a model constructed to distinguish between invasive and non-invasive lesions including only mean CT value reached 0.808.

The present radiomics signatures consisted of five radiomics features. Root mean squared (RMS) is first-order histogram feature. It also remained in radiomics model in the study by Weng et al. (33) and in the study by She et al. (29). RMS is related to the characteristics of the intensity distribution in the pulmonary nodules. Both dependence entropy and large dependence high gray level emphasis are gray level dependence matrix features which indicate the relationship between the gray-level intensity of CT voxels and the invasiveness of GGNs. The higher value indicated more heterogeneity in the texture patterns. Wavelet.LHL_gldm_DependenceEntropy and gradient_ glszm_ZoneEntropy are radiomics features undergoing image transformation with a filter. Both are calculated from gray-level intensity features. The higher values of these features in the IAC group meant that IAC was more heterogeneous than AIS/MIA. The radiomics signatures including four gray level related features showed that the gray-level intensity value might be of importance in predicting the risk of IAC of GGNs.

A radiomics model aiming to diagnose IAC in the study by Wu et al. (34) had a higher AUC of 0.98. One reason might be the study included only part-solid nodules. The radiomics model combined ground-glass and solid features. In addition, the larger diameter of the pulmonary nodules in that study might be another reason. Even so, there are some limitations in this study. First, the ratio of the IAC group and the AIS/MIA group was consistent with the actual clinical scenario. We didn’t selectively collect the samples to balance the two groups, so the imbalanced sample ratio of IAC and AIS/MIA may have had an impact on the nomogram model. Second, the CT images in this study came from four different CT scanner, which may cause potential variability because of different parameters. Third, the reconstruction matrix of 512*512 for small GGNs may limit the diagnosis ability of radiomics. Scanning and reconstruction of local regions of the target images can reduce the size of pixels and increase the information of segmented areas of small GGNs, thus improving the diagnosis ability. Higher pixel matrix, such as 1024*1024 or 2048*2048 could break the limitation of CT image reconstruction matrix and improve the diagnosis ability. Last, the data collection is retrospective, a larger prospective longitudinal cohort is needed to confirm the performance of our nomogram model.

In summary, this study presents a nomogram incorporating radiomics features and radiological features of CT images to predict the risk of IAC in patients with GGNs measuring 5-10mm in diameter. The nomogram can serve as a potential tool to guide individual diagnosis and help clinician choose the optimal intervention.

## Data Availability Statement

The raw data supporting the conclusions of this article will be made available by the authors, without undue reservation.

## Ethics Statement

The studies involving human participants were reviewed and approved by The Institutional Review Board of Shanghai Public Health Clinical Center, Fudan University and Affiliated Hospital of Nantong University. Written informed consent for participation was not required for this study in accordance with the national legislation and the institutional requirements.

## Author Contributions

LS: the acquisition of data, analysis of data, and drafting the article. WS: the acquisition of data and the interpretation of data. XP: the analysis of data. YZ: the acquisition of data. LZ: the acquisition of data. YW: the acquisition of data. MF: the acquisition of data. JZ: the acquisition of data. FS: the conception and design of the study, revising the article, and final approval of the version to be submitted. LL: the conception of the study, revising the article, and final approval of the version to be submitted. All authors contributed to the article and approved the submitted version.

## Funding

This work was supported by the National Natural Science Foundation of China (No.91846302) and the Shanghai Municipal Commission of Health and Family Planning, China (No. 2018ZHYL0104).

## Conflict of Interest

The authors declare that the research was conducted in the absence of any commercial or financial relationships that could be construed as a potential conflict of interest.
