# Supplementary material for: Development and Validation a Nomogram Incorporating CT Radiomics Signatures and Radiological Features for Differentiating Invasive Adenocarcinoma From Adenocarcinoma In Situ and Minimally Invasive Adenocarcinoma Presenting as Ground-Glass Nodules Measuring 5-10mm in Diameter
Source: Front Oncol. 2021 Apr 21;11:618677. doi: 10.3389/fonc.2021.618677 (PMC8096901; doi:10.3389/fonc.2021.618677)
Supplement: Supplementary file 1 [file DataSheet_1.docx]

**Supplementary files**

**Radiomics features extraction**

A total of 1525 features were calculated for each voxel setting using a combination of open source PyRadiomics software (https://pyradiomics.readthedocs.io/en/latest/index.html) and in-house code. The original image has 111 features. Each image that underwent square, square root, logarithm, exponential, gradient and local binary pattern(lbp) in 2D filter has 101 features. Three dimensional wavelet transform was applied to the volume of the interest. L and H is low-pass and high-pass filtering. The wavelet decompositions of volume can be labeled as wavelet_LLL, wavelet _LLH, wavelet _LHL, wavelet _LHH, wavelet _HLL, wavelet _HLH, wavelet _HHL and wavelet _HHH. For example, wavelet_LLH is obtained from x-directional low-pass filtering, y-directional low-pass filtering and z-directional high-pass filtering of volume of interest. Each wavelet decomposition has 101 features. Nine shape 3D features (voxel volume, compactness 1, compactness 2, spherical disproportion, major axis length, minor axis length, least axis length, elongation and flatness) were not obtained from the filtered images. So far, 1525 radiomics features were obtained.

Table 1 The original features classification and the corresponding feature names

| Original features classification | Feature names |
| --- | --- |
| First Order Statistics (19 features) | Energy, Total Energy, Entropy, Minimum, 10th Percentile, 90th Percentile, Maximum, Mean, Median, Interquartile Range, Range, Mean Absolute Deviation, Robust Mean Absolute Deviation, Root Mean Squared, Standard Deviation, Skewness, Kurtosis, Variance, Uniformity |
| Shape-based (3D) (17 features) | Mesh Volume, Voxel Volume, Surface Area, Surface Area to Volume ratio, Sphericity, Compactness 1, Compactness 2, Spherical Disproportion, Maximum 3D diameter, Maximum 2D diameter (Slice), Maximum 2D diameter (Column), Maximum 2D diameter (Row), Major Axis Length, Minor Axis Length, Least Axis Length, Elongation, Flatness |
| Gray Level Cooccurence Matrix （24 features） | Autocorrelation, Joint Average, Cluster Prominence, Cluster Shade, Cluster Tendency, Contrast, Correlation, Difference Average, Difference Entropy, Difference Variance, Joint Energy, Joint Entropy, Informational Measure of Correlation 1, Informational Measure of Correlation 2, Inverse Difference Moment, Maximal Correlation Coefficient, Inverse Difference Moment Normalized, Inverse Difference, Inverse Difference Normalized, Inverse Variance, Maximum Probability, Sum Average, Sum Entropy, Sum of Squares |
| Gray Level Size Zone Matrix (16 features) | Small Area Emphasis, Large Area Emphasis, Gray Level Non-Uniformity(size zone), Gray Level Non-Uniformity Normalized(size zone), Size Zone Non-Uniformity, Size Zone Non-Uniformity Normalized, Zone Percentage, Gray Level Variance, Zone Variance, Zone Entropy, Low Gray Level Zone Emphasis, High Gray Level Zone Emphasis, Small Area Low Gray Level Emphasis, Small Area High Gray Level Emphasis, Large Area Low Gray Level Emphasis, Large Area High Gray Level Emphasis |
| Gray Level Run Length Matrix (16 features) | Short Run Emphasis, Long Run Emphasis, Gray Level Non-Uniformity, Gray Level Non-Uniformity Normalized, Run Length Non-Uniformity, Run Length Non-Uniformity Normalized, Run Percentage, Gray Level Variance, Run Variance, Run Entropy, Low Gray Level Run Emphasis, High Gray Level Run Emphasis, Short Run Low Gray Level Emphasis, Short Run High Gray Level Emphasis, Long Run Low Gray Level Emphasis, Long Run High Gray Level Emphasis |
| Neighbouring Gray Tone Difference Matrix (5 features) | Coarseness, Contrast, Busyness, Complexity, Strength |
| Gray Level Dependence Matrix (14 features) | Small Dependence Emphasis, Large Dependence Emphasis, Gray Level Non-Uniformity, Dependence Non-Uniformity, Dependence Non-Uniformity Normalized, Gray Level Variance, Dependence Variance, Dependence Entropy, Low Gray Level Emphasis, High Gray Level Emphasis, Small Dependence Low Gray Level Emphasis, Small Dependence High Gray Level Emphasis, Large Dependence Low Gray Level Emphasis, Large Dependence High Gray Level Emphasis |

**Radiomics features and formulas**

**The rad-score of each ground-glass nodule (GGN) was calculated via the linear combination of selected features using the formulas as follows:**

-7.326+0.479*wavelet.LHL_gldm_DependenceEntropy

+0.411*gradient_glszm_ZoneEntropy

+8.779e-05*Large_Dependence_High_Gray_Level_Emphasis

+0.508*Dependence_Entropy

-3.450e-03*Root_Mean_Squared.

The formula was presented like a logistic regression formula, such as

$\text{y=}\beta_{0} +\beta_{1}x1 +\beta_{2}x2 +\beta_{3}x3 +\beta_{4}x4+\beta_{5}x5$. The number -7.326 indicated the*β*_0_, the number 0.411 indicated the*β*_1_, * indicated the multiplication sign, **and the value of** wavelet.LHL_gldm_DependenceEntropy indicated the *x*_1_. 8.779e-05 and 3.450e-03 was presented as scientific notation.
