# Supplementary figures and images for: Development and Validation a Nomogram Incorporating CT Radiomics Signatures and Radiological Features for Differentiating Invasive Adenocarcinoma From Adenocarcinoma In Situ and Minimally Invasive Adenocarcinoma Presenting as Ground-Glass Nodules Measuring 5-10mm in Diameter
Source: Front Oncol. 2021 Apr 21;11:618677. doi: 10.3389/fonc.2021.618677 (PMC8096901; doi:10.3389/fonc.2021.618677)

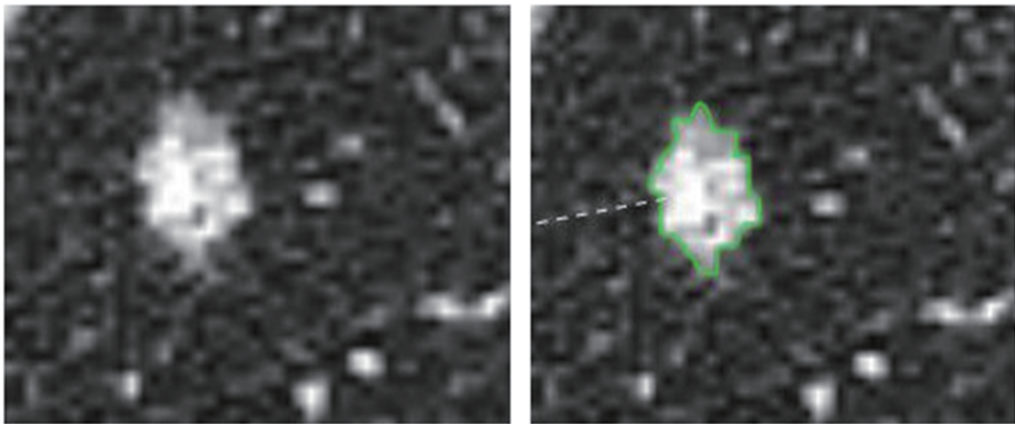

Figure 1 A slice example of the nodule segmentation

Supplement: Supplementary file 2 [file Image_1.pdf]
